# Effects of an asymmetrical high flow nasal cannula interface in hypoxemic patients

**DOI:** 10.1186/s13054-023-04441-6

**Published:** 2023-04-18

**Authors:** Douglas Slobod, Elena Spinelli, Stefania Crotti, Alfredo Lissoni, Alessandro Galazzi, Giacomo Grasselli, Tommaso Mauri

**Affiliations:** 1 Department of Anesthesia, Critical Care and Emergency, Fondazione IRCCS Ca’ Granda, Ospedale Maggiore Policlinico, Via F. Sforza 35, 20122 Milan, Italy; 2 Department of Critical Care Medicine, McGill University, Montreal, Canada; 3 Department of Healthcare Professions, Fondazione IRCCS Ca’ Granda Ospedale Maggiore Policlinico, Milan, Italy; 4 Department of Pathophysiology and Transplantation, University of Milan, Milan, Italy

**Keywords:** High flow nasal cannula, Ventilatory efficiency, Asymmetrical interface, Electrical impedance tomography, Esophageal pressure monitoring, Hypoxemic respiratory failure

## Abstract

**Background:**

Optimal noninvasive respiratory support for patients with hypoxemic respiratory failure should minimize work of breathing without increasing the transpulmonary pressure. Recently, an asymmetrical high flow nasal cannula (HFNC) interface (Duet, Fisher & Paykel Healthcare Ltd), in which the caliber of each nasal prong is different, was approved for clinical use. This system might reduce work of breathing by lowering minute ventilation and improving respiratory mechanics.

**Methods:**

We enrolled 10 patients ≥ 18 years of age who were admitted to the Ospedale Maggiore Policlinico ICU in Milan, Italy, and had a PaO_2_/FiO_2_ < 300 mmHg during HFNC support with a conventional cannula. We investigated whether the asymmetrical interface, compared to a conventional high flow nasal cannula, reduces minute ventilation and work of breathing. Each patient underwent support with the asymmetrical interface and the conventional interface, applied in a randomized sequence. Each interface was provided at a flow rate of 40 l/min followed by 60 l/min. Patients were continuously monitored with esophageal manometry and electrical impedance tomography.

**Results:**

Application of the asymmetrical interface resulted in a −13.5 [−19.4 to (−4.5)] % change in minute ventilation at a flow rate of 40 l/min, *p* = 0.006 and a −19.6 [−28.0 to (−7.5)] % change at 60 l/min, *p* = 0.002, that occurred despite no change in PaCO_2_ (35 [33–42] versus 35 [33–43] mmHg at 40 l/min and 35 [32–41] versus 36 [32–43] mmHg at 60 l/min). Correspondingly, the asymmetrical interface lowered the inspiratory esophageal pressure–time product from 163 [118–210] to 140 [84–159] (cmH_2_O*s)/min at a flow rate of 40 l/min, *p* = 0.02 and from 142 [123–178] to 117 [90–137] (cmH_2_O*s)/min at a flow rate of 60 l/min, *p* = 0.04.

The asymmetrical cannula did not have any impact on oxygenation, the dorsal fraction of ventilation, dynamic lung compliance, or end-expiratory lung impedance, suggesting no major effect on PEEP, lung mechanics, or alveolar recruitment.

**Conclusions:**

An asymmetrical HFNC interface reduces minute ventilation and work of breathing in patients with mild-to-moderate hypoxemic respiratory failure supported with a conventional interface. This appears to be primarily driven by increased ventilatory efficiency due to enhanced CO_2_ clearance from the upper airway.

**Supplementary Information:**

The online version contains supplementary material available at 10.1186/s13054-023-04441-6.

## Introduction

High flow nasal cannula (HFNC) is the recommended first-line noninvasive respiratory support for patients with acute hypoxemic respiratory failure [1–3]. Despite its simple design, HFNC is associated with clinically relevant physiological benefits, promotes lung and diaphragm protection, and potentially allows clinicians to avoid endotracheal intubation [4]. Several mechanisms have been extensively described: greater concordance between set FiO_2_ and alveolar PO_2_ improves oxygenation; positive end-expiratory pressure (PEEP) effect increases end-expiratory lung volume [5]; and CO_2_ washout from the upper airway decreases minute ventilation [5, 6]. All may increase patient comfort compared to standard, low flow oxygen therapy. Although the presence and magnitude of these physiological effects vary from patient to patient, their combination decreases excessive respiratory drive. The final clinically relevant mechanisms being 1) decreased inspiratory effort and risk of diaphragmatic injury [7] and 2) decreased transpulmonary driving pressure and risk of patient self-inflicted lung injury [8].

However, the percentage of patients failing HFNC support is still high [9, 10]. Failure of HFNC leads to escalation of noninvasive support (to helmet continuous positive airway pressure and/or noninvasive positive pressure ventilation) and admission to the ICU. Delayed intubation after HFNC failure is associated with increased mortality [11]. Thus, simple methods to increase the efficacy of HFNC support would be a welcome addition to our treatments to avoid failure and/or admission to the ICU. Recently, we described additional physiological benefits conferred by HFNC delivered at flows higher than 60 l/min, but patient comfort was poor, and sedation might be required to facilitate this approach [12]. Alternatively, awake prone positioning during support with HFNC could further increase oxygenation [13–15], maximize lung protection [16], and decrease work of breathing [17]. However, prone positioning requires active patient collaboration and might not be feasible in postoperative respiratory failure and severely obese patients.

Recently, a novel HFNC interface that uses an asymmetrical cannula design was approved for clinical use. The asymmetrical cannula features one prong with a smaller diameter and one prong with a larger diameter, resulting in an overall 30–40% increase in the total cross-sectional area compared to an equally sized conventional interface [18]. Only bench studies have been conducted so far, demonstrating that the asymmetrical configuration potentially increases the positive airway pressure effect and enhances CO_2_ washout compared to a conventional symmetrical cannula [18, 19]. Airway pressure may be increased with the asymmetrical interface due to the overall greater total cross-sectional area of the prongs, resulting in a greater prong area-to-nare area ratio [6] and CO_2_ washout may be augmented due to the creation of a pressure difference between the nasal cavities and a pattern of reverse flow from the more occluded to the less occluded nare [18].

In the present study, we investigated the impact of the asymmetrical cannula on minute ventilation, work of breathing and the underlying physiological mechanisms in spontaneously breathing patients with acute mild-to-moderate hypoxemic respiratory failure.

## Methods

This was a single-center, prospective, physiologic, crossover study of patients admitted to the intensive care unit (ICU) of the Ospedale Maggiore Policlinico in Milan, Italy. Patients ≥ 18 years of age were eligible for inclusion if they were admitted to the ICU due to an acute respiratory condition starting within the prior 7 days and a PaO_2_/FiO_2_ < 300 mmHg while already supported with a conventional HFNC interface. Exclusion criteria included pregnancy, respiratory acidosis of any origin (PaCO_2_ > 45 mmHg and pH < 7.30), presence of neuromuscular disease, and contraindications to esophageal pressure monitoring (uncontrolled coagulopathy, nasal trauma, esophageal disease). The study protocol was approved by the research ethics board of the Ospedale Maggiore Policlinico (Ref. no. 930_2022).

After obtaining informed consent, baseline demographic and clinical data were collected, and an esophageal balloon catheter (Cooper Surgical, Trumbull, USA) was inserted and calibrated according to the presence of cardiac oscillations and negative deflections during inspiration [5, 20]. Then, a 16-electrode electrical impedance tomography (EIT) belt was placed around the chest at the fifth or sixth intercostal space and connected to a bedside EIT monitor. Esophageal pressure (Pes) and EIT data were recorded simultaneously at 20 Hz and stored on a dedicated data acquisition system (Dräger PulmoVista® 500, Lübeck, Germany) for offline analysis.

The study protocol consisted of application of the asymmetrical interface (Optiflow Duet, Fisher and Paykel Healthcare, Auckland, New Zealand) and the conventional interface, in a randomized sequence, for 30 min each. The size of the conventional interface was the one in place for clinical use at study enrollment. The asymmetrical interface was sized identically to the conventional interface in place at enrollment (i.e., large or medium size for both). No instructions were provided to patients regarding mouth opening during breathing.

Each interface was provided at a flow rate of 40 l/min for 15 min followed by a flow rate of 60 l/min for 15 min (4 total steps with randomization of cannula type). An oxygen saturation (SpO_2_) of 92–96% was maintained throughout the study by adjusting the set FiO_2_. In the last 3–5 min of each step, we recorded hemodynamic and respiratory parameters, Pes and EIT data, assessed dyspnea, and obtained arterial blood gas analyses in patients with an arterial cannula. Tidal volume (V_T_) was estimated from the tidal change in impedance using a conversion factor as in our previous work [21].

The primary endpoint was the change in minute ventilation in patients supported with the asymmetrical cannula interface compared to the standard interface at each flow rate. Secondary endpoints included the impact on work of breathing, quantified as the simplified inspiratory pressure time product per minute (PTP_min_), calculated from the esophageal pressure waveform, and the following physiological parameters, chosen to identify potential mechanisms explaining the effects of the asymmetrical interface:

### Gas exchange

1. PaCO_2_
2. SpO_2_/FiO_2_
3. PaO_2_/FiO_2_

### Lung mechanics

1. Dynamic lung compliance computed as V_T_ / ∆Pes [17]
2. Regional dynamic lung compliance (ventral and dorsal)
3. End-expiratory lung volume analyzed as the end-expiratory lung impedance
4. The percentage of ventilation reaching the dependent half of the lungs (dorsal fraction of ventilation)

### Indicators of respiratory drive

1. Mean inspiratory flow computed as V_T_ / inspiratory time
2. Modified Borg dyspnea scale [22]
3. Ratio of oxygen saturation (ROX) index [10]
4. Respiratory rate

We also investigated whether the asymmetrical cannula had any effect on hemodynamics by evaluating the following:

### Hemodynamics

1. Systolic blood pressure
2. Mean arterial blood pressure
3. Heart rate

### Statistical Analysis

Based on prior work [5] and a predicted 20% change in minute ventilation considered as clinically relevant, a sample size of 8 patients was selected to attain a probability of type-I error of 0.05 and a statistical power of 80%. The sample size was increased to 10 patients to account for potential missing data (i.e., poor quality of EIT recordings). EIT data were obtained and analyzed in all patients, Pes data were obtained from 8 patients, and arterial blood gas analyses were obtained in 5 patients.

Descriptive statistics were used to characterize the study population. Continuous data are presented as median and interquartile range [IQR]. Comparisons between interfaces were analyzed at both flow rates using the Wilcoxon signed rank test. A *p*-value of < 0.05 was considered statistically significant.

To investigate whether baseline clinical or physiological characteristics predicted the magnitude of reduction in minute ventilation after application of the asymmetrical cannula, we performed simple linear regression analysis of relevant predictors and the percentage change in minute ventilation resulting from application of the asymmetrical cannula at each flow rate. We considered the following predictors: PaO_2_/FiO_2_, number of quadrants affected on chest radiograph, and Sequential Organ Failure Assessment (SOFA) score at enrollment, Simplified Acute Physiology Score (SAPS II) score at ICU admission, respiratory rate, baseline minute ventilation and mean inspiratory flow (respiratory drive) measured during HFNC support with the conventional interface at 40 l/min. When a significant association was identified for a given predictor, we performed a linear mixed-effects analysis that accounted for the repeated-measures design across flow rates. The percentage change in minute ventilation was defined as the dependent variable, flow rate and the predictor were considered fixed-effects variables, and individual patients were treated as a random-effects variable.

## Results

Data from 10 patients were analyzed and their baseline demographics, and clinical information is presented in Table 1. Patients were majoritarily male and had a SAPS II score of 41 [37–47] upon ICU admission. At enrollment, the PaO_2_/FiO_2_ was 249 [187–258] mmHg and 2 [1–4] quadrants were affected on chest radiography.

**Table 1:** Baseline demographics and clinical information

Patient	Sex	Age (year)	SAPS II at ICU admission	SOFA acore at enrollment	PaO_2_/setFiO_2_ (mmHg)	PaCO_2_ (mmHg)	Co-morbidities	Etiology of acute respiratory failure	Quadrants affected on chest radiograph
1	M	50	40	6	176	40	None	Primary, infectious	4
2	M	48	33	2	248	31	Cardiovascular disease	Primary, infectious	4
3	F	71	52	3	190	31	Cardiovascular disease	Primary, non-infectious	2
4	M	76	39	3	200	39	Cardiovascular disease, malignancy	Primary, infectious	4
5	F	74	64	4	250	42	Cardiovascular disease, malignancy, venous thromboembolic disease	Primary, infectious	1
6	M	62	38	3	172	41	Chronic pulmonary disease, cardiovascular disease	Extrapulmonary, non-infectious	1
7	F	86	43	7	249	34	Cardiovascular disease	Extrapulmonary, infectious	4
8	M	38	45	7	250	53	None	Extrapulmonary, non-infectious	1
9	M	75	41	6	282	36	Cardiovascular disease	Primary, infectious	2
10	M	33	26	2	285	32	Hematologic malignancy	Extrapulmonary, non-infectious	2
Total or median [IQR]	3F / 7 M	67 [46–75]	41 [37–47]	4 [3–6]	249 [187–258]	38[32–41]	7 cardiovascular disease, 1 chronic pulmonary disease, 3 malignancy, 1 venous thromboembolic disease	6 primary, 4 extrapulmonary, 7 infectious, 3 non-infectious	2 [1–4]

Data are presented as median [IQR]. Definition of abbreviations: IQR = interquartile range, SOFA = Sequential Organ Failure Assessment, SAPS = Simplified Acute Physiology Score

**Fig. 1:**
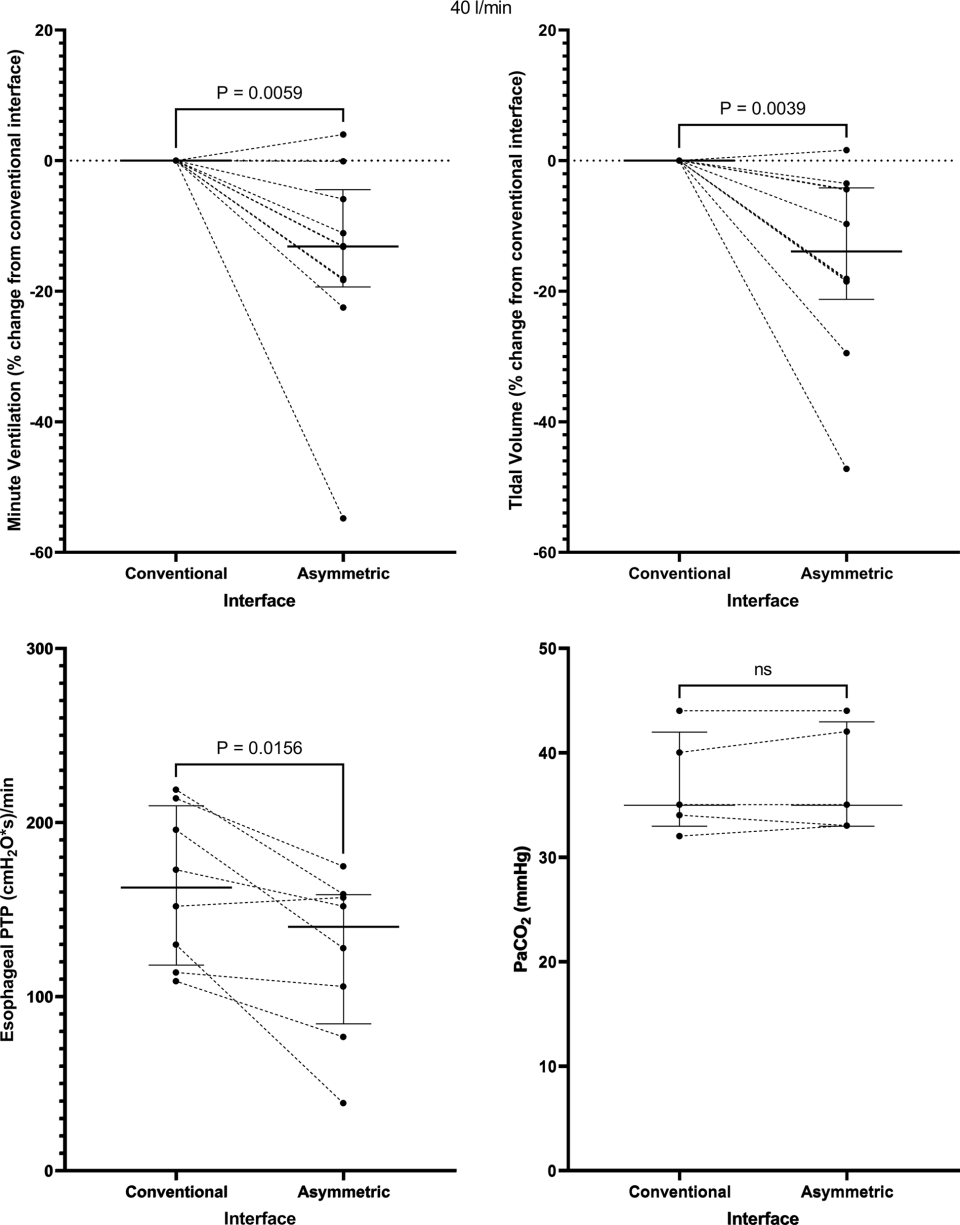
At a flow rate of 40 l/min, the asymmetrical cannula lowered minute ventilation, tidal volume, and the inspiratory esophageal pressure–time product (an indicator of the metabolic work of breathing over 1 min) despite no change in the arterial carbon dioxide tension. PTP = pressure–time product. Horizontal bars represent median and interquartile range

**Fig. 2:**
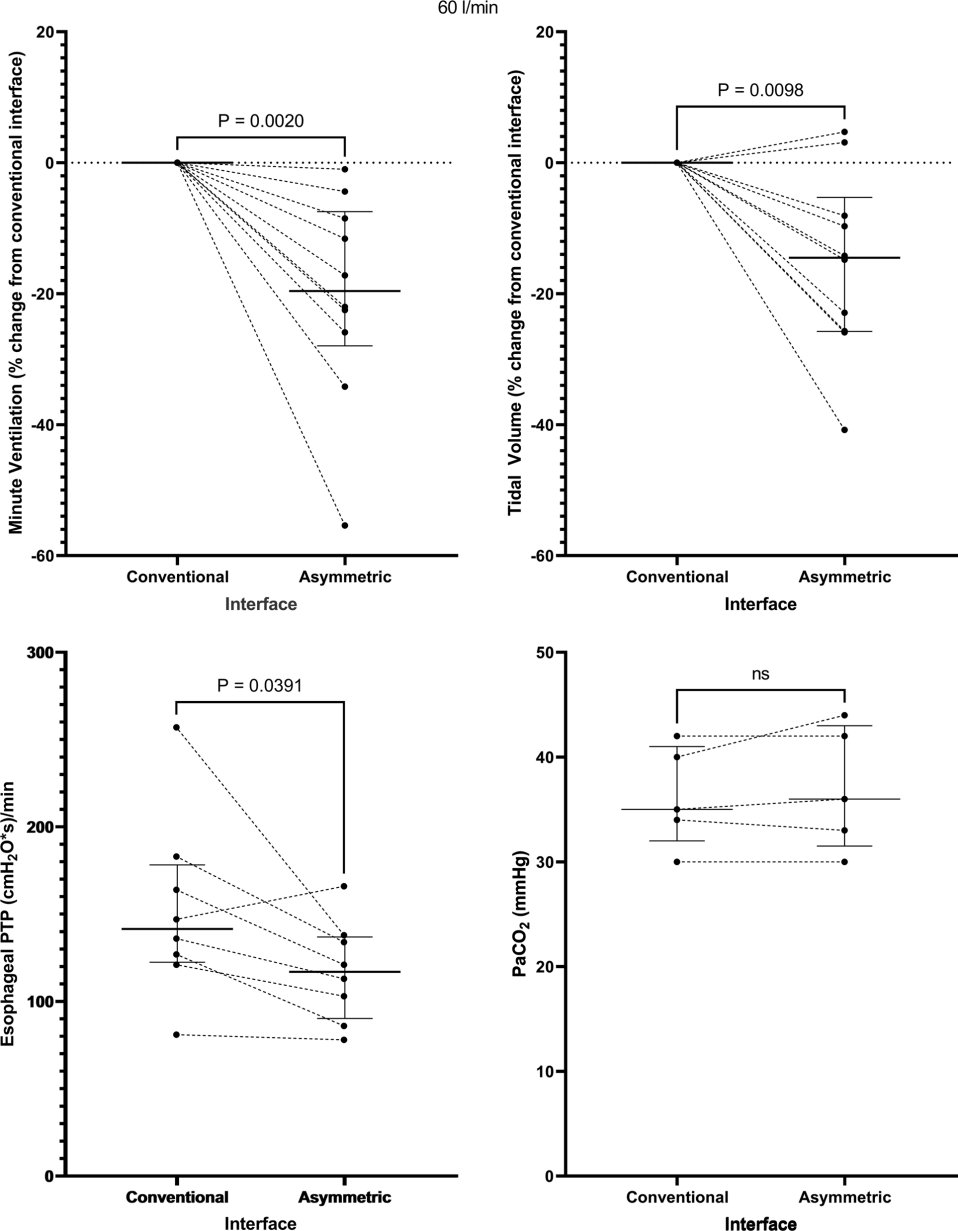
Findings at a flow rate of 60 l/min: the asymmetrical cannula lowered minute ventilation, tidal volume, and the inspiratory esophageal pressure–time product despite no change in the arterial carbon dioxide tension. PTP = pressure–time product. Horizontal bars represent median and interquartile range

Application of the asymmetrical interface resulted in a decrease in the minute ventilation from 10.9 [7.4–13.7] to 9.7 [6.3–10.8] l/min at a flow rate of 40 l/min, *p* = 0.01 and from 9.4 [6.5–14.7] to 8.5 [4.8–10.8] l/min at a flow rate of 60 l/min, *p* = 0.002. This corresponded to a -13.5 [−19.4 to (−4.5)] % change in minute ventilation at a flow rate of 40 l/min, *p* = 0.006 (Fig. 1) and a −19.6 [−28.0 to (−7.5)] % change at 60 l/min, *p* = 0.002 (Fig. 2). The change in minute ventilation was mediated by a decrease in tidal volume (512 [356–647] to 401 [336–600] ml at a flow rate of 40 l/min, *p* = 0.004 and 468 [320–662] to 396 [244–569] ml at 60 l/min, *p* = 0.02) that occurred despite no change in PaCO_2_ (35 [33–42] versus 35 [33–43] mmHg at 40 l/min and 35 [32–41] versus 36 [32–43] mmHg at 60 l/min) (Figs. 1 and 2, Table 2).

**Table 2:** Impact of the asymmetrical interface on inspiratory effort and work of breathing

	40 l/min		*p* Value	60 l/min		*p* value
	Conventional	Asymmetrical		Conventional	Asymmetrical	
*Respiratory parameters*						
Minute Ventilation (l/min)	10.9 [7.4–13.7]	9.7 [6.3–10.8]	0.01	9.4 [6.5–14.7]	8.5 [4.8–10.8]	0.002
Minute Ventilation (% change from conventional interface)	–	−13.2 [−19.4 to (-4.5)]	0.006	–	−19.6 [−28.0 to (−7.5)]	0.002
V_T_ (ml)	512 [356–647]	401 [336–600]	0.004	468 [320–662]	396 [244–569]	0.02
V_T_ (% change from conventional interface)	–	−13.9 [−21.3 to (−4.2)]	0.004	–	−14.5 [−25.8 to (−5.3)]	0.01
Respiratory rate (breaths per minute)	22 [20–26]	21 [18–26]	0.81	24 [18–27]	21 [18–26]	0.20
*Effort*						
∆Pes (cmH_2_O)	10.1 [8.1–11.9]	8.9 [7.1–11.0]	0.64	9.8 [7.0–12.8]	8.3 [6.5–11.7]	0.15
Inspiratory Esophageal PTP/min (cmH_2_O*s/min)	163 [118–210]	140 [84–159]	0.02	142 [123–178]	117 [90–137]	0.04
*Lung mechanics*						
Dynamic lung compliance (ml/cmH_2_O)	48 [38–86]	50 [38–80]	0.93	51 [42–101]	54 [30–67]	0.82
EELZ (Au)	1648 [1059–3454]	1847 [1211–2611]	0.85	1249 [772–2487]	1861 [1225–2138]	0.38
Dorsal Fraction of Ventilation (%)	63 [51–70]	66 [48–70]	0.84	62 [49–68]	62 [47–68]	0.99
Ventral dynamic lung compliance (ml/cmH_2_O)	19 [14–27]	16 [14–28]	0.77	20 [14–43]	17 [15–24]	0.92
Dorsal dynamic lung compliance (ml/cmH_2_O)	32 [22–60]	29 [22–54]	0.98	36 [21–58]	33 [19–50]	0.73
*Gas exchange*						
PaO_2_ (mmHg)	86 [79–91]	86 [80–98]	0.75	94 [84–106]	94 [85–104]	0.88
PaCO_2_ (mmHg)	35 [33–42]	35 [33–43]	0.75	35 [32–41]	36 [32–43]	0.75
pH	7.46 [7.46–7.48]	7.46 [7.45–7.51]	0.50	7.48 [7.44–7.49]	7.45 [7.45–7.49]	> 0.99
FiO_2_ (%)	40 [30–41]	40 [30–47]	0.50	40 [30–44]	40 [31–46]	0.50
SpO_2_/FiO_2_ (%)	250 [238–322]	250 [210–320]	0.34	250 [221–322]	250 [212–315]	0.06
PaO_2_/FiO_2_ (mmHg)	227 [198–292]	216 [196–285]	0.13	252 [213–310]	233 [204–315]	0.63
*Hemodynamics*						
Systolic Blood Pressure (mmHg)	126 [108–154]	129 [98–138]	0.08	126 [108–147]	132 [94–137]	0.04
Mean Arterial Pressure (mmHg)	96 [74–103]	81 [68–97]	0.07	94 [73–100]	85 [69–94]	0.05
Heart Rate (beats per minute)	90 [73–103]	82 [74–102]	0.11	92 [72–105]	87 [72–97]	0.25
*Respiratory drive*						
Borg dyspnea scale	2 [0–3]	1 [0–2]	0.63	1 [0–4]	1 [0–2]	0.25
Mean Inspiratory Flow (l/min)	25.0 [18.8–38.3]	20.5 [17.8–30.8]	0.07	24.5 [15.8–39.3]	22.0 [12.5–28.25]	0.02
ROX Index	14 [9–16]	14 [9–16]	0.84	13 [9–16]	13 [9–16]	0.84

Data are presented as median [IQR]

IQR = interquartile range, V_T_ = tidal volume, EELZ = end-expiratory lung impedance, Au = arbitrary units, ∆Pes = change in esophageal pressure, PTP = pressure–time product

Correspondingly, application of the asymmetrical interface lowered the inspiratory esophageal pressure–time product (an index of the metabolic work of breathing over 1 min) from 163 [118–210] to 140 [84–159] (cmH_2_O*s)/min at a flow rate of 40 l/min, *p* = 0.02 and from 142 [123–178] to 117 [90–137] (cmH_2_O*s)/min at a flow rate of 60 l/min, *p* = 0.04 (Figs. 1 and 2). Interestingly, 2 patients who manifested no change or an increase in minute ventilation seen in Fig. 1 also developed a decrease in esophageal pressure–time product, suggesting that minute ventilation might not always be an accurate reflection of work of breathing. There was a trend toward a decrease in respiratory rate (Additional file 1: Fig. S1) and the change in Pes (∆Pes) (Additional file 1: Fig. S2) that did not reach statistical significance at each flow rate.

**Fig. 3:**
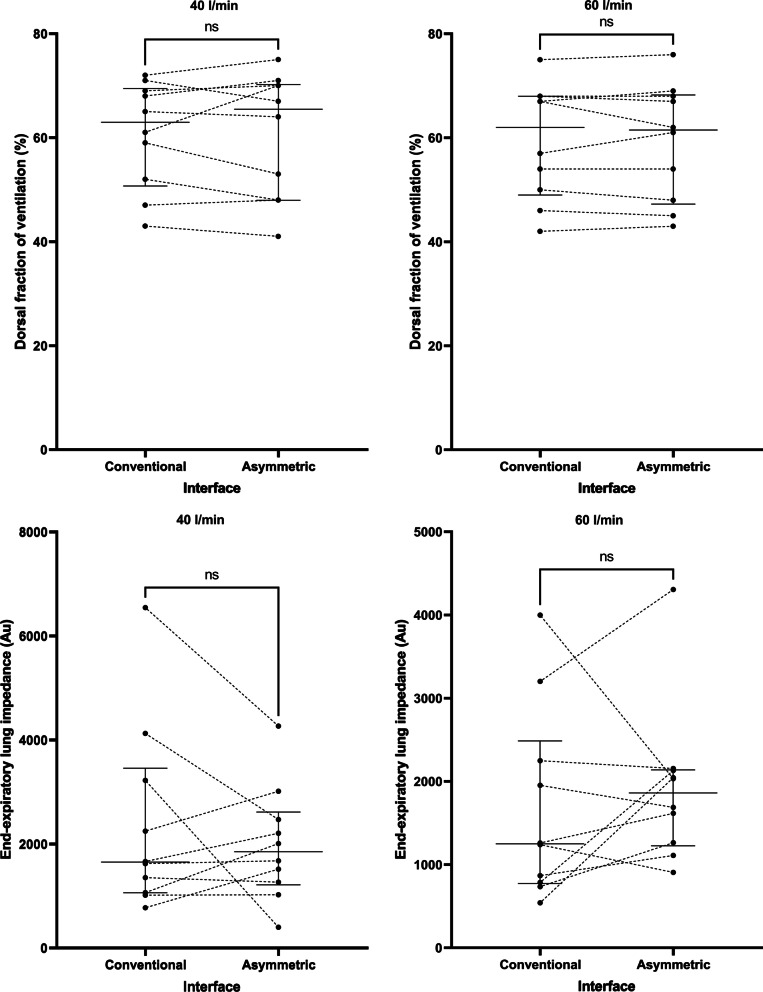
Impact of the asymmetrical cannula interface on lung mechanics at both delivered flow rates. Au = arbitrary units. Horizontal bars represent median and interquartile range

Application of the asymmetrical cannula did not have any impact on average values of oxygenation, the dorsal fraction of ventilation, global or regional dynamic lung compliance, or end-expiratory lung impedance (Table 2), suggesting that there was no major effect on PEEP, lung mechanics, or alveolar recruitment at a flow rate of 40 l/min or 60 l/min (Fig. 3). Notably, 4 patients (40%) did manifest an increase in end-expiratory lung volume with the asymmetrical interface at 40 l/min (Fig. 3) and 6 patients (60%) at 60 l/min (Fig. 3), suggesting that a PEEP effect may be present in specific patient subgroups (e.g., extrapulmonary lung injury). There was no effect on patient reported dyspnea, and the asymmetrical interface was well tolerated overall.

**Fig. 4:**
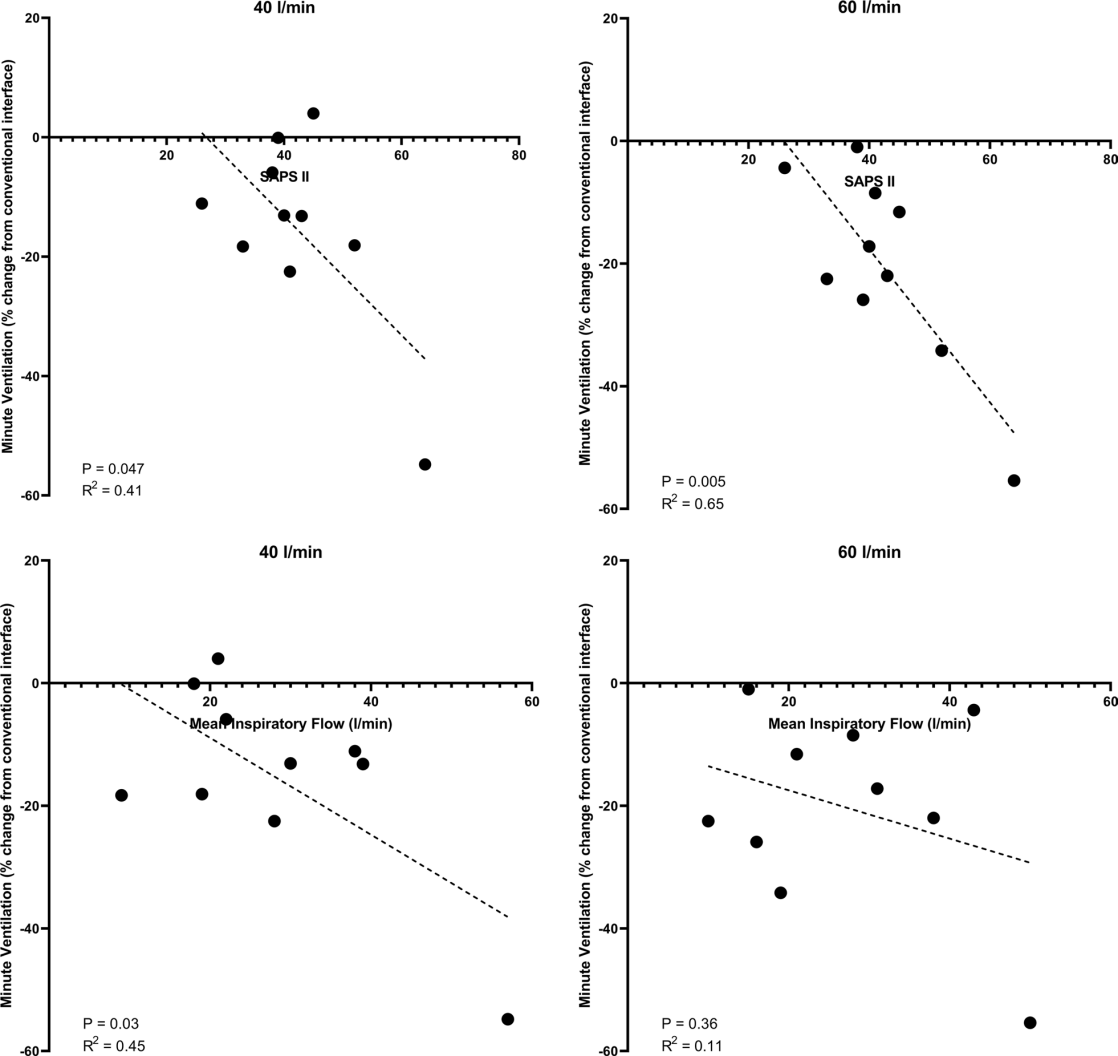
Correlations between SAPS II and mean inspiratory flow and the change in minute ventilation following application of the asymmetrical interface at each flow rate

Of the tested clinical and physiological predictors, higher SAPS II score at ICU admission was significantly associated with a greater percentage decrease in minute ventilation conferred by the asymmetrical interface at both flow rates (Fig. 4), whereas higher minute ventilation (R^2^ = 0.46, *p* = 0.03) and mean inspiratory flow during support with the conventional interface were associated with a greater decrease in minute ventilation at a flow rate of 40 l/min (Fig. 4). Linear mixed-effects analysis demonstrated that every 1-point increase in SAPS II at admission was associated with a −1.1 [−1.9 to (−0.38)] percentage point change in minute ventilation (Table 3) suggesting that greater severity of illness at ICU admission was associated with a greater reduction in minute ventilation following application of the asymmetrical cannula. Linear mixed-effects analyses of baseline minute ventilation and mean inspiratory flow did not demonstrate a significant relationship with the change in minute ventilation.

**Table 3:** Results from a linear mixed-effects analysis of the impact of SAPS II on the percentage change in minute ventilation following application of asymmetrical interface

	Estimate	95% CI	*p* Value
Intercept	27.0	−5.2 to 59.1	0.09
Flow rate 40 l/min	5.0	−3.6 to 3.6	0.22
Flow rate 60 l/min	–	–	–
SAPS II	−1.1	−1.9 to (−0.4)	0.008

## Discussion

The main finding of our study is that application of an asymmetrical high flow nasal cannula interface in patients with mild-to-moderate hypoxemia who are already supported with a conventional interface is associated with a reduction in minute ventilation and work of breathing despite no change in PaCO_2_, indicating increased ventilatory efficiency. End-expiratory lung inflation and alveolar recruitment, on average, were not improved by the asymmetrical interface suggesting absence of large PEEP effect compared to the conventional interface.

These results are consistent with findings recently reported by Tatkov et al. who performed a study of the asymmetrical interface in an upper airway model [18]. They demonstrated improved dead-space clearance and more efficient CO_2_ elimination from the upper airway with the asymmetrical interface that was due to the preferential flow of gas out of the lesser occluded nare and redirected flow from the larger cannula to the contralateral nare during expiration. Similar findings were also recently reported by Vieira et al. in a bench study comparing a conventional interface to the asymmetrical interface [19]. They demonstrated a reduction in rebreathing volume from the upper airway with the asymmetrical interface that was generally amplified at higher flow rates. The potential benefits for patients being decreased dyspnea and work of breathing.

Our data demonstrate that the asymmetrical interface was not associated with any change in oxygenation or lung mechanics, suggesting that the overall mechanism of the reduction in work of breathing may not be due to a mean airway pressure effect on lung recruitment. This finding differs from data presented in previous studies. Studying symmetrical interfaces of varying size, Pinkham et al. demonstrated that a greater prong area-to-nare area ratio resulted in greater end-expiratory airway pressure in a bench model and that this effect was magnified at higher flow rates [23]. Tatkov et al. reported higher PEEP during support with the asymmetrical interface, particularly at flow rates of 40 and 60 l/min with a maximal difference of 2–3 cmH_2_O [18]. Vieira et al. also reported higher nasopharyngeal pressure at end expiration with the asymmetrical cannula. The difference in pressure was more pronounced with increasing flow rate and respiratory rate. The authors of both studies attributed this difference to increased airflow resistance induced by the asymmetrical interface, both during inspiration and expiration [19, 24]. We did not measure end-expiratory airway pressure directly and cannot corroborate these findings. A lack of evidence supporting an airway pressure effect in our study may be explained by a lack of recruitability in this patient population or by an increase in airway pressure too small to be clinically relevant. Another possibility is that we did not instruct patients not to breathe with the mouth open, a common occurrence in awake patients. We, therefore, cannot make a conclusion regarding the effect of the asymmetrical interface on PEEP. However, if in some patients, PEEP was increased, the potential physiological benefits of the asymmetrical cannula could be even greater.

The finding that a greater severity of illness at ICU admission, assessed with the SAPS II score, might predict a greater reduction in minute ventilation following application of the asymmetrical cannula suggests that patients with a greater burden of systemic disease may derive physiologic benefits from support with the asymmetrical interface. This complements our prior study that demonstrated that support with HFNC was effective at reducing respiratory drive and work of breathing in patients with extrapulmonary sepsis, over 50% of whom had a diagnosis of septic shock with a median SAPS II score of 37 at admission to the ICU [25]. In patients with more severe systemic disease and increased CO_2_ production, enhanced CO_2_ clearance by the asymmetrical cannula could be even more clinically relevant. However, our finding should also be considered in the context of a recent retrospective study of 200 patients with COVID-19, in whom a greater SAPS II score was associated with HFNC failure, defined as a need for intubation following admission to the ICU [11] and a study of 202 onco-hematology patients who received HFNC support in the ICU for acute respiratory failure, in whom a greater SAPS II score was associated with increased risk of intubation and hospital mortality after HFNC failure [26]. These studies underscore the clinical need for a HFNC device with increased physiological effects in patients with a greater severity of illness.

The exploratory finding that higher minute ventilation and mean inspiratory flow (V_T_ / inspiratory time), reflecting higher respiratory drive, during support with the conventional interface may indicate a patient who will manifest a greater reduction in minute ventilation and work of breathing with the asymmetrical interface may help clinicians in tailoring their approach to selecting patients most likely to benefit from the asymmetrical interface. Previous data have shown that higher PaCO_2_ during support with low flow oxygen therapy predicted a greater reduction in the tidal change in Pes following support with HFNC [5].

Overall, we observed that the impact of the asymmetrical interface was greatest when it was applied at 60 l/min. Compared to the conventional interface applied at 40 l/min, the asymmetrical interface applied at 60 l/min resulted in a minute ventilation that was > 2 l/min lower. Despite our patients having a normal PaCO_2_ and pH during support with the conventional interface, enhanced CO_2_ clearance by the asymmetrical interface may have assisted patients in maintaining a normal PaCO_2_ with unchanged respiratory drive at a HFNC flow rate of 40 l/min and decreased respiratory drive at 60 l/min [27, 28]. Reducing elevated respiratory drive and breathing effort while maintaining lung recruitment and gas exchange represent key objectives of a lung and diaphragm-protective ventilation strategy [7].

Our study has limitations: First a small number of patients were studied and only 5 had measurements of PaCO_2_ available at each step, rendering conclusions regarding ventilatory efficiency hypothesis generating; second, all patients had mild-to-moderate hypoxemia without significant dyspnea, limiting the generalizability of the findings to a broader critically ill and hypercapnic population; third, we did not control for the potential impact of open mouth breathing, a variable that has a significant impact on the development of PEEP during support with HFNC [24]; fourth, we did not measure gastric pressures or assess for expiratory muscle recruitment that could have led to an overestimation of ∆Pes and the esophageal pressure–time product; and fifth, we estimated V_T_ and minute ventilation based on EIT derived measurements of the tidal change in lung impedance. However, this method has been used in previous studies [25] and has increased validity given that patients are compared with themselves across HFNC interface steps.

## Conclusion

An asymmetrical HFNC interface reduces minute ventilation and work of breathing in patients with mild-to-moderate hypoxemic respiratory failure supported with a conventional high flow nasal cannula. This appears to be primarily driven by increased ventilatory efficiency due to enhanced CO_2_ clearance from the upper airway.

## Supplementary Information

**Additional file 1**: **Fig. S1**. Respiratory rate before and after application of the asymmetric high flow nasal cannula interface. The difference was not significant at each flow rate (see Table 2 in main manuscript for statistical information). Horizontal bars represent median and interquartile range. **Fig. S2**. The change in esophageal pressure (∆Pes) before and after application of the asymmetric high flow nasal cannula interface. The difference was not significant at each flow rate (see Table 2 in main manuscript for statistical information). Horizontal bars represent median and interquartile range.

## Data Availability

The datasets used and analyzed in the current study are available from the corresponding author on reasonable request.

## Abbreviations

HFNC: High flow nasal cannula
PEEP: Positive end-expiratory pressure
ICU: Intensive care unit
EIT: Electrical impedance tomography
Pes: Esophageal pressure
∆Pes: Change in esophageal pressure
V_T_: Tidal volume
PTP_min_: Inspiratory esophageal pressure time product per minute
ROX index: Ratio of oxygen saturation index
SOFA: Sequential organ failure assessment
SAPS II: Simplified acute physiology score
∆Z: Impedance change
