# Supplementary material for: Effects of an asymmetrical high flow nasal cannula interface in hypoxemic patients
Source: Crit Care. 2023 Apr 18;27:145. doi: 10.1186/s13054-023-04441-6 (PMC10111067; doi:10.1186/s13054-023-04441-6)

**Effects of an Asymmetrical High Flow Nasal Cannula Interface in Hypoxemic Patients: Online Supplement**

Douglas Slobod, Elena Spinelli, Stefania Crotti, Alfredo Lissoni, Alessandro Galazzi, Giacomo Grasselli, Tommaso Mauri

**Supplementary Figure 1:** Respiratory rate before and after application of the asymmetric high flow nasal cannula interface. The difference was not significant at each flow rate (see Table 2 in main manuscript for statistical information). Horizontal bars represent median and interquartile range.


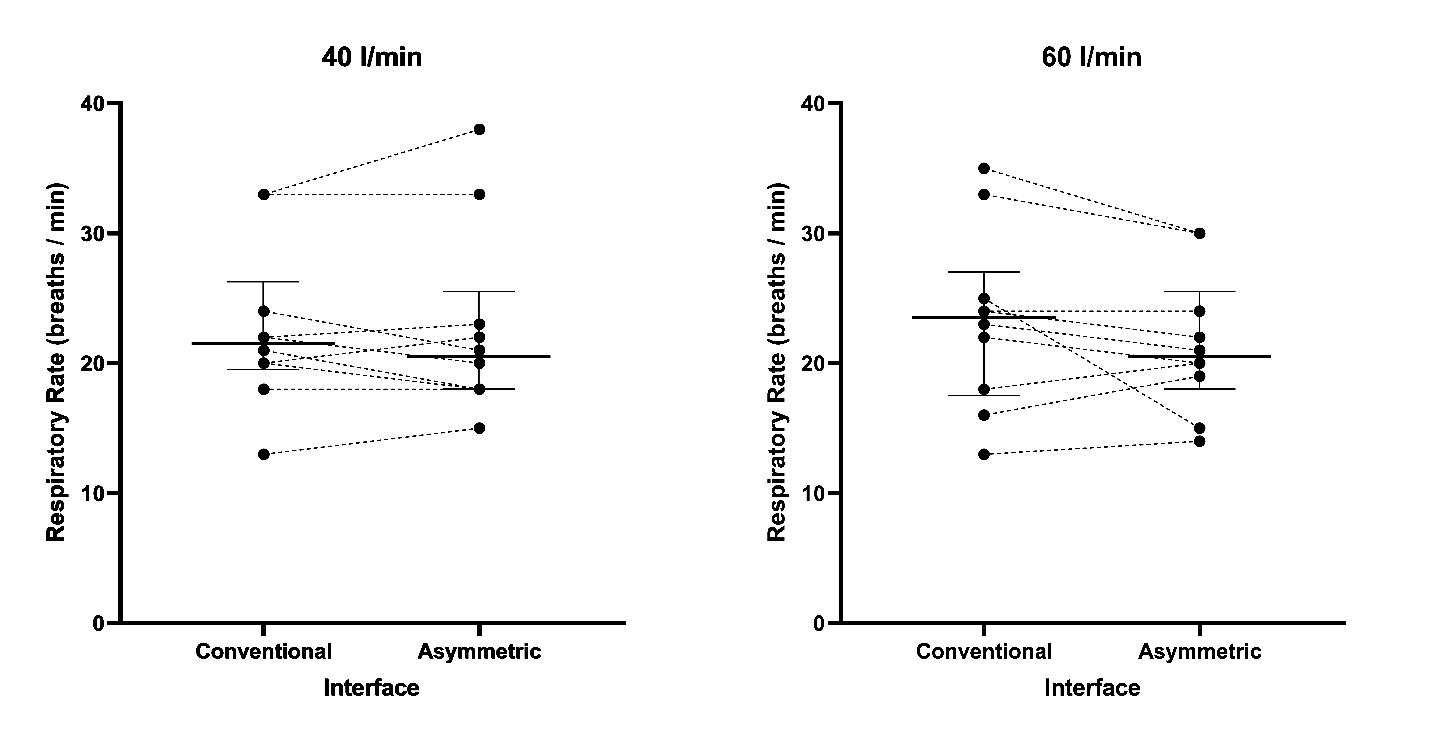


**Supplementary Figure 2:** The change in esophageal pressure (∆Pes) before and after application of the asymmetric high flow nasal cannula interface. The difference was not significant at each flow rate (see Table 2 in main manuscript for statistical information). Horizontal bars represent median and interquartile range.


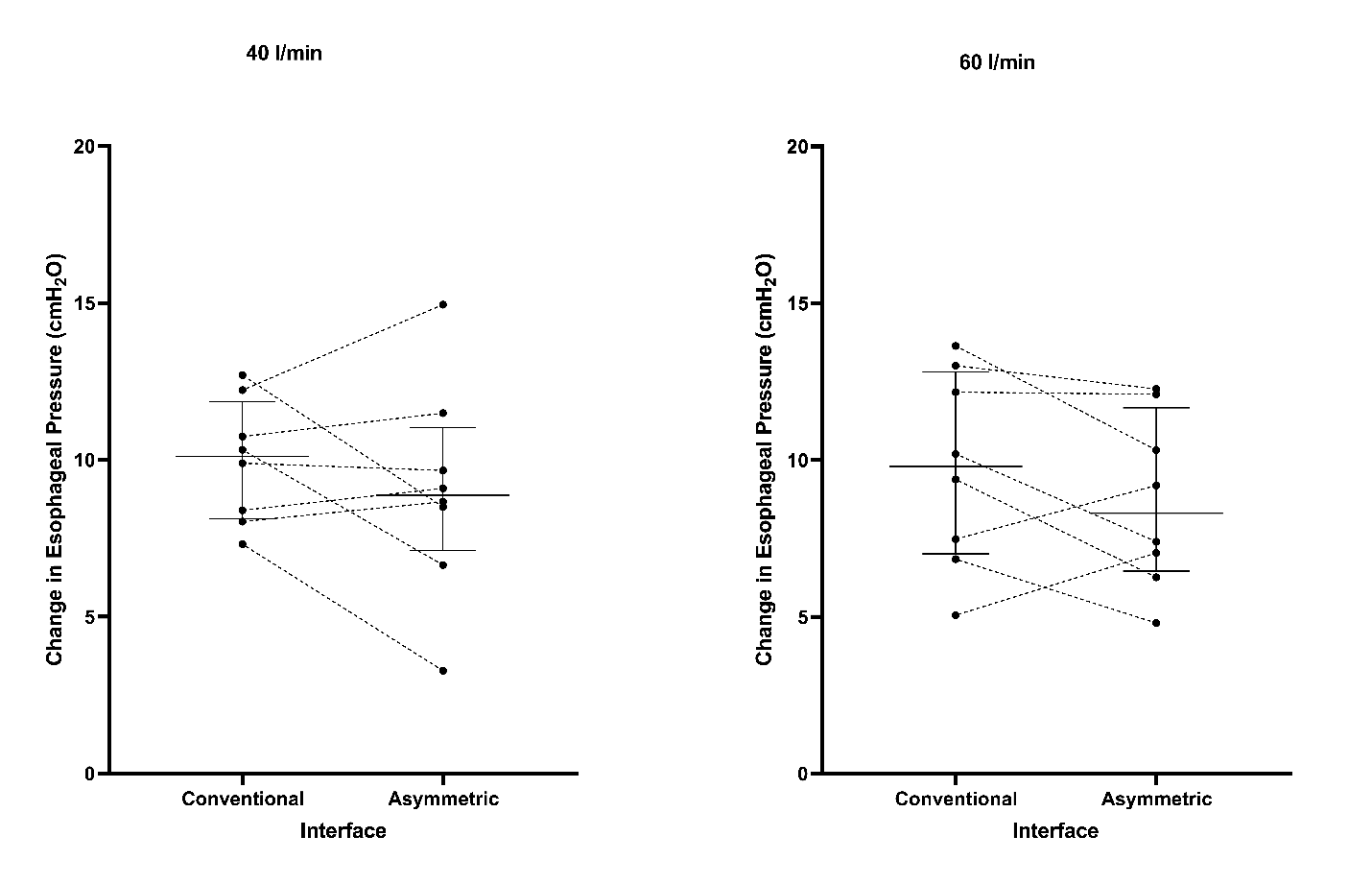

Supplement: Supplementary file 1 — Additional file 1: Fig. S1. Respiratory rate before and after application of the asymmetric high flow nasal cannula interface. The difference was not significant at each flow rate (see Table 2 in main manuscript for statistical information). Horizontal bars represent median and interquartile range. Fig. S2. The change in esophageal pressure (∆Pes) before and after application of the asymmetric high flow nasal cannula interface. The difference was not significant at each flow rate (see Table 2 in main manuscript for statistical information). Horizontal bars represent median and interquartile range. [file 13054_2023_4441_MOESM1_ESM.docx]
